# Dr. Guiseppe Lapponi

**Published:** 1907-01

**Authors:** 


Dr. Guiseppe Lapponi, physician to Pope Pius X, died at Rome, December 7, 1906, aged about 45 years. His disease was cancer of the stomach, but the immediate cause of his death was pneumonia, which set in a few days before his death.

